# Focal glial activation coincides with increased BACE1 activation and precedes amyloid plaque deposition in APP[V717I] transgenic mice

**DOI:** 10.1186/1742-2094-2-22

**Published:** 2005-10-07

**Authors:** Michael T Heneka, Magdalena Sastre, Lucia Dumitrescu-Ozimek, Ilse Dewachter, Jochen Walter, Thomas Klockgether, Fred Van Leuven

**Affiliations:** 1 Department of Neurology, University of Münster, 48149 Münster, Germany; 2 Department of Neurology, University of Bonn, 53127 Bonn, Germany; 3 Experimental Genetics Group, Dept Human Genetics, K.U.Leuven, B-3000 Leuven, Belgium

## Abstract

**Background:**

Inflammation is suspected to contribute to the progression and severity of neurodegeneration in Alzheimer's disease (AD). Transgenic mice overexpressing the london mutant of amyloid precursor protein, APP [V717I], robustly recapitulate the amyloid pathology of AD.

**Methods:**

Early and late, temporal and spatial characteristics of inflammation were studied in APP [V717I] mice at 3 and 16 month of age. Glial activation and expression of inflammatory markers were determined by immunohistochemistry and RT-PCR. Amyloid deposition was assessed by immunohistochemistry, thioflavine S staining and western blot experiments. BACE1 activity was detected in brain lysates and in situ using the BACE1 activity kit from R&D Systems, Wiesbaden, Germany.

**Results:**

Foci of activated micro- and astroglia were already detected at age 3 months, before any amyloid deposition. Inflammation parameters comprised increased mRNA levels coding for interleukin-1β, interleukin-6, major histocompatibility complex II and macrophage-colony-stimulating-factor-receptor. Foci of CD11b-positive microglia expressed these cytokines and were neighbored by activated astrocytes. Remarkably, β-secretase (BACE1) mRNA, neuronal BACE1 protein at sites of focal inflammation and total BACE1 enzyme activity were increased in 3 month old APP transgenic mice, relative to age-matched non-transgenic mice. In aged APP transgenic mice, the mRNA of all inflammatory markers analysed was increased, accompanied by astroglial iNOS expression and NO-dependent peroxynitrite release, and with glial activation near almost all diffuse and senile Aβ deposits.

**Conclusion:**

The early and focal glial activation, in conjunction with upregulated BACE1 mRNA, protein and activity in the presence of its substrate APP, is proposed to represent the earliest sites of amyloid deposition, likely evolving into amyloid plaques.

## Background

Alzheimer's disease (AD) is a neurodegenerative disorder that is characterized by progressive memory loss and decline of cognitive functions. Histopathological hallmarks include extracellular amyloid peptide (Aβ) deposition in neuritic plaques, and intracellular deposits of hyperphosphorylated Tau, causing formation of neurofibrillary tangles and finally neuronal death. Aβ peptides are generated from amyloid precursor protein (APP) by sequential actions of two proteolytic enzymes, i.e. the β-site APP cleavage enzyme (BACE1) and the γ-secretase [1,2]. Their formation and eventual deposition represents a key feature and possibly the triggering mechanism of AD. The importance of Aβ formation was instigated by dominantly inherited familial forms of AD that are linked to APP mutations in or close to the β- and γ-secretase cleavage sites [3]. This made it possible to generate transgenic mouse models of cerebral amyloidosis and AD-like histopathology, i.e. amyloid plaques and cerebral amyloid angiopathy (CAA) [4-6](3–8) [7,8].

The eventual deposition of Aβ and the neurofibrillary tangle formation may not account for all, and particularly not for the most early clinical symptoms in AD. Inflammatory changes are observed in AD brain overall, and particularly at the amyloid depots, invariably comprising activated microglia [9,10]. Once stimulated by beginning neuronal degeneration, microglia releases, a wide variety of pro-inflammatory mediators including cytokines, complement components, various free radicals and nitric oxide (NO), which all contribute to further neuronal dysfunction and eventually death. These create and feed a vicious cycle that could be essential in the pathological progression of AD [11]. Apart from any direct effects of microglial inflammation, the recruitment of astrocytes that assemble around and in amyloid plaques are likely to prolong the ongoing inflammation.

In addition to histopathological and biochemical data, several proinflammatory genes have been linked to an increased risk for AD, including interleukin1 (Il-1) [12], interleukin 6 (Il-6) [13] and tumor necrosis factor alpha (TNFα) [14]. The hypothesis that inflammatory changes actively contribute to AD pathogenesis is further supported by epidemiological data, i.e. long term medication with non-steroidal anti-inflammatory drugs (NSAIDs) appears to decrease the risk, delay the onset and slow the cognitive decline of AD patients [15-17].

The finding that cytokines are able to transcriptionally upregulate BACE1 mRNA, protein and enzyme activity levels and thereby increase total and fibrillogenic Aβ peptides in cell-biological models [18] prompted us to test the hypothesis that BACE1 is related to age-dependent parameters of inflammation in vivo, i.e. in the brain of APP [V717I] transgenic mice. The data presented are an important extension of the phenotypic characterization of APP [V717I] mice which recapitulate not only the amyloid [6] and cerebrovascular angiopathy [7] but various aspects of neuroinflammation. Moreover, they indicate that early and focal inflammation may feedback stimulate local APP processing via BACE1 and these sites therefore possibly represent the birthplaces of plaques.

## Methods

### Animals

Transgenic mice expressing APP [V717I] under the mouse thy1 gene promoter in the FVB/N genetic background [6] aged 3 and 16 months were used in this study with non-transgenic mice of the same genetic background, gender and age as controls. At the time of sacrifice, animals were anesthetised and transcardially perfused with heparinized sodium chloride (0.9%), brains were removed and several regions including frontal cortex and cerebellum dissected from one hemisphere using the mouse brain atlas coordinates [19]. Dissected sections were snap frozen in liquid nitrogen and stored at -80°C until analysis. The remaining hemisphere was fixed either in 4% paraformaldehyde followed by paraffin embedding or underwent cryofixation under tissue protection with tissue frezzing medium (Leica Instruments, Nussloch, Germany) according to standard protocols, before sectioning for immunohistochemistry. Animal care and handling was performed according to the declaration of Helsinki and approved by local ethical committees (approval #50.203.2BN 33,34/00).

### Immunohistochemistry

Serial sagittal sections were cut (7 μm) from parrafin embedded tissue (Leica microtome RM2155) and mounted (Histobond adhesion slides, Marienfeld, Germany). Retrieval of antigen sites, blocking of endogenous peroxidase activity and blocking of non-specific binding sites was performed according to standard protocols. For immunostaining of paraffin-embedded tissue, sections were incubated overnight at 4°C with the following primary antibodies: 1) mouse mAb against GFAP, #MAB360 (1:800, Chemicon, Hofheim, Germany). 2) rabbit pAb against iNOS, 32030 (1:150, Transduction Laboratories, Heidelberg, Germany). 3) rabbit pAb against Aβ1–42, #44–344 (1:40, Biosource International, USA.). Immunohistochemical localization was performed using the avidin-biotin peroxidase complex method (ABC-Kit, Vector Laboratories, Burlingame, USA) with 3,3'-diaminobenzidine tetrahydrochloride as chromogen. For costaining in paraffin tissue of GFAP and Aβ1–42, slides were washed twice in PBS and blocked in 20% normal goat serum. After incubation with the primary antibody for 20 h slides were washed and incubated with biotinylated goat anti rabbit IgG. Immunohistochemical localisation was detected as described above using Vector-blue as substrate (Vector-blue substrate kit, Vector Laboratories, Burlingame, USA).

All other single or double immunostaining was performed on cryofixed sections cut (6 μm) and mounted as described above. Sections were dried at RT for 1 h and then fixed in 4% PFA or methanol for 15 min at RT. After washing with PBS the double staining was performed by adding simultaneously both first antibodies and followed by overnight incubation at 4°C. In addition to the above decribed antibodies the following antibodies were used: 4) rat mAb #MCA 711 against murine CD11b (CD11b, 1:250, Serotec Düsseldorf, Germany). 5) rat mAb against Il-1β, MAB401 (1:50, R&D Systems, Wiesbaden-Nordenstadt, Germany). 6) goat pAb against IL 6, M12 sc1265 (1:200, Santa Cruz, Biotechnology Inc., Heidelberg, Germany). 7) 7520 rabbit pAb against the C-terminal domain of BACE1 (gift from Dr. Christian Haass, Adolf-Butenandt-Institute, University of Munich). 8) mouse mAb anti nitrotyrosine # 05–233 (1:40, Upstate Inc., Biomol, Hamburg) 9) rabbit pAb GFAP, Z334 against glial fibrillary acidic protein (1:800, DAKO, Hamburg, Germany). 10) mouse mAb # MAB 377 against neuronal nuclei (neuN, 1:500, Chemicon, Hofheim, Germany). The goat secondary antibodies (Fluorescein DTAF conjugated anti rabbit 1:150, Texas Red conjugated anti mouse 1:80, Texas Red conjugated anti rat 1:80, Jackson Immuno Research Laboratories, West Grove, USA) were applied sequentially after washing in PBS. Negative controls included non-specific IgG instead of primary antibodies; pre-absorption with respective cognate peptides (150–200 μg of peptide/ml of antibody working solution), omission of the secondary antibody and absence of immunoreactivity in non-transgenic controls of the respective age.

### Confocal laser scanning microscopy

Double-labeled specimens were analyzed with a confocal laser scanning microscope (Multiprobe 2001; Molecular Probes, Inc., Eugene, OR) equipped with an Ar/Kr laser with balanced emission at 488, 568, and 647 nm. Images were aquired at a 40 × magnification to ensure a high quality resolution of microglial cells. To achieve an optimal signal-to-noise ratio for each fluorophore, sequential scanning with 568 and 488 nm was used. The digitalized images were then processed with ImageSpace 3.10 software (Molecular Probes, Inc.) on a Silicon Graphics (Mountain View, CA) power series 310GTX work station. Original section series were subjected to Gaussian filtration to reduce noise and enhance weakly but specifically labeled parts. Original and filtered sections were projected on one plane using a maximum-intensity algorithm and in some cases using depth-coding and surface-rendering algorithms.

### Thioflavine-S staining

Thioflavine-S staining consisted of reacting section in 0.015% aqueous thioflavine-S for 10 min, followed by differentiation in 50% ethanol, rinsing in water, air draining and clarification into xylene. Thereafter slides were covered and evaluated under fluorescent lighting using UV filtration and a standard microscope (Nikon, Eclipse E-800).

### Quantification of immunohistochemistry

For quantitative image analysis of hippocampal and cortical immunostaining, serial sagittal sections taken from lateral (+0.5–+2.25) were examined. iNOS, GFAP and CD11b staining cells as well as Aβ1–42-positive neuritic plaques were counted on sections of 6 animals per group. Antigens were detected in 10 parallel sections with defined distance of 70 μm showing both the hippocampus and cortex. In each section, 20 randomly choosen fields were evaluated. Cell number was determined using a counting grid at 20 × magnification and given as calculations of square millimeters. Images were aquired using a standard light and immunofluorescence microscope (Nikon, Eclipse E-800) connected to a digital camera (SONY, model DXC-9100P, Köln, Germany) and to a PC system with LUCIA imaging software (LUCIA 32G, version 4.11; Laboratory Imaging, Düsseldorf, Germany). Data were analysed by ANOVA with Tukey's post test using SYSTAT (Systat, Evanston, U.S.A.).

### RNA preparation and RT-PCR

Brain sections from frontal cortex and cerebellum were dissected and RNA extracted from using Trizol reagent as recommended by the manufacturer (Sigma, St. Louis, MO), followed by RT-PCR. The primers were: iNOS forward 5'-TGGGAGCCACAGCAATATAG-3' and iNOS reverse 5'-ACAGTTTGGTGTGGTGTAGG-3'; GFAP forward 5'-TCCGCGGCACGAACGAGTC-3' and GFAP reverse 5'-CACCATCCCGCATCTCCACAGTCT-3'; MCSF-R forward 5'-GACCTGCTCCACTTCTCCAG-3' and MCSF-R reverse 5'-GGGTTC AGACCAAGCGAGAAG-3'; MHCII forward 5'-CTGATGGCTGCTCATCCTGTGC-3' and MHCII reverse 5'-TTCTGTTTTCTGTATGCTGTCC-3'; IL-1β forward 5'-CCTGTGTAATGAAAGACGGC-3' and IL-1β reverse 5'-AAGGGA GCTCCTTCACA TGC-3'; GAPDH forward 5'-TCACCAGGGCTGCCATTTGC-3' and GAPDH reverse 5'-GACTCCACGACATACTCAGC-3'; IL-6 forward 5'- CAGAAA CCGCTATGAAGT TCC-3' and IL-6 reverse 5'-TGTACTCCAGGTAGCTATGG-3'. TGF-β1 forward 5'-CAAGTGTGGAGCAACATGTG-3' and TGFβ-1 reverse 5'-CACAGCAGTTCTTCTCT GTG-3', BACE1 forward 5'-CCGGCG GGAGTGG TATTATGAAGT-3' and BACE1 reverse 5'GATGGTGATGCGGAAGGACTGATT-3'. PCRs were carried out on RNA from n = 6 animals in each group, and representative gels of 2 animals per group are shown. PCR conditions were 35 cycles (iNOS, GFAP, IL-1β, TGF-β1, IL-6, MHCII, MCSF-R, BACE1) and 24 cyles (GAPDH) of denaturation at 95°C for 30s; annealing at 63°C for 45s, and extension at 72°C for 45s using a PX2 (ThermoHybaid, Ulm, Germany). PCR products were separated by electrophoresis through 2% agarose containing 0.5 μg/ml ethidium bromide and imaged using an AlphaInotech imaging system (Temeculah, USA).

### Determination of Aβ

Frontal cortex from transgenic mice were homogenized in RIPA buffer (1% Triton, 1% sodium deoxycholate, 0.1% SDS, 150 mM NaCl, 50 mM Tris-HCl, pH 7.2) using an Ultraturrax T25 (Janke&Kunkel, IKA-Labortechnik). Aβ was immunoprecipitated from 100 μg protein using antibody 2964 and protein A beads (Amersham Pharmacia, Freiburg, Germany), separated on 10–20% Tris tricine gels (Anamed, Darmstadt, Germany) and transferred onto nitrocellulose membranes. Aβ was detected by immunoblotting with antibody 6E10 (Signet labs Inc, Dedham, MA).

### Determination of BACE activity

The enzymatic activity of BACE1 was measured in membrane extracts from frontal cortex by fluorimetric reaction as suggested by the supplier (BACE activity kit FP002, R&D Systems, Wiesbaden, Germany). In addition, BACE1 activity was determined *in situ *using serial cryosections. Sections were stored at -70°C and immediately before analysis kept at -20°C for 15 min and 4°C for 10 min. Thereafter sections were incubated at 4°C in PBS plus 0.4% TritonX for 30 min. After addition of 5 μl of fluorgenic BACE1 substrate and 100 μl of 1x substrate buffer, sections were incubated at 37°C for 1 hr. Then, sections were rinsed in PBS and mounted with Mowiol4-88 (Calbiochem, San Diego, CA, USA). BACE1 activity was visualized using a DAPI filter set (Ex. 340–380, Emis:435-485) and a standard light and immunofluorescence microscope (Nikon, Eclipse E-800) connected to a digital camera (SONY, model DXC-9100P, Köln, Germany) and to a PC system with LUCIA imaging software (LUCIA 32G, version 4.11; Laboratory Imaging, Düsseldorf, Germany). Addition of a BACE1 inhibitor served as control as previously described [20]. Parallel sections were used to detect GFAP immunostaining as described above. Computational overlay analysis was employed to estimate the colocalisation of BACE1 activity/GFAP expression.

### Quantification of RT-PCR and immunoblot results

RT-PCR was quantified by densitometry of at least 6 animals per age. Band intensities were determined using Image-J software (NIH). Data were analyzed by ANOVA with Tukey's post test (Systat, Evanston, U.S.A.).

## Results

**Figure 1:**
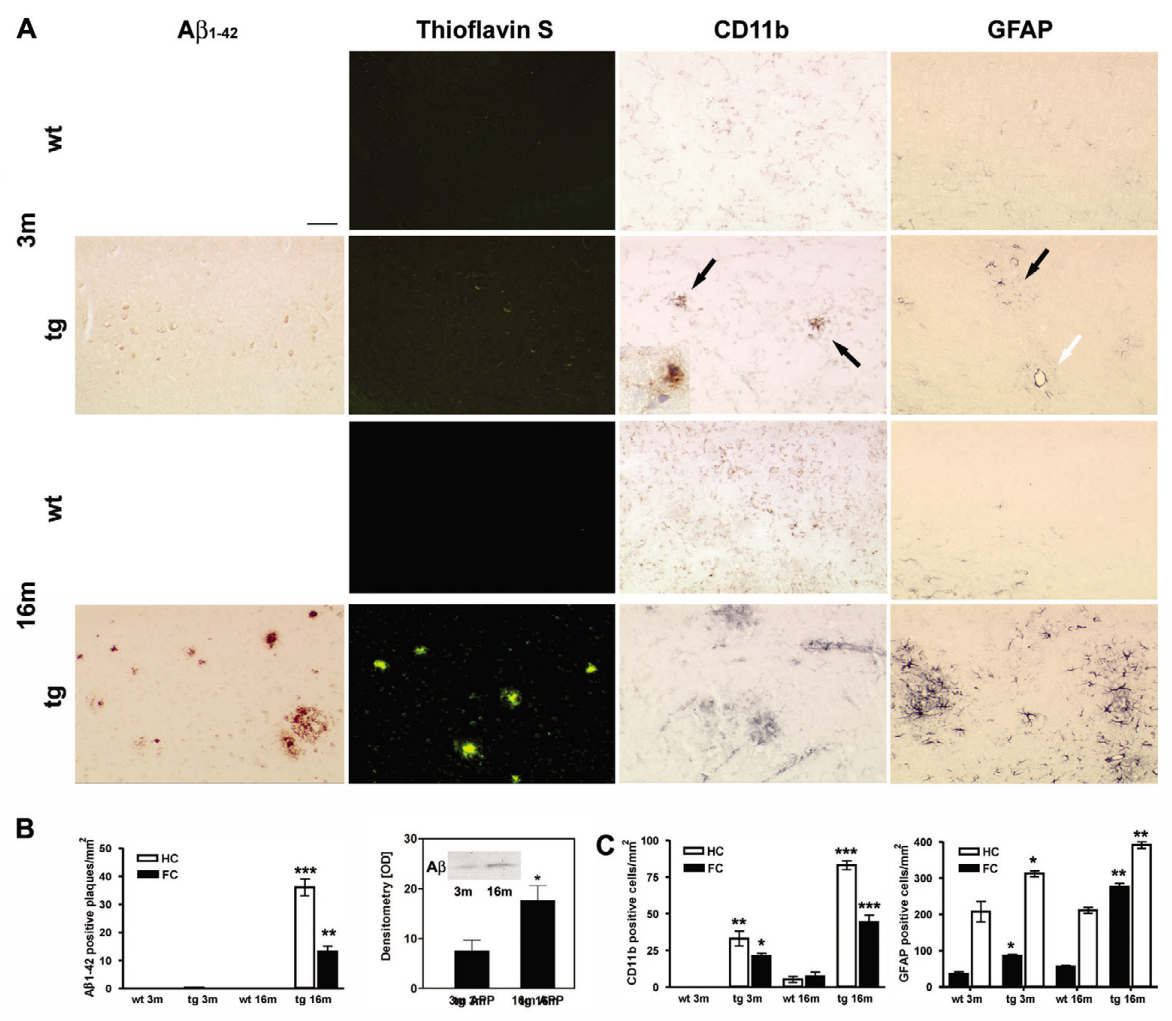
**Comparison of Aβ deposition, micro- and astroglial activation. **(**A**) Representative detection of Aβ1–42 immunostaining, Thioflavin-S histochemistry, microglial (CD11b) and astroglial activation (GFAP) in APP [V717I] mice and non-transgenic controls of the identical genetic background at 3 (3 m) and 16 (16 m) months (Bar graph = 50 μm (Aβ1–42, Thioflavin-S), = 25 μm (CD11b, GFAP)) Focal microglial activation is indicated by black arrows. Focal astroglial activation within the parenchyma by black arrows and at the side of of a brain vessel by a white arrow (**B**). Quantification of hippocampal (HC, open bar) and cortical (FC, filled bar) Aβ1–42-positive plaques of APP [V717I] mice at 3 and 16 months (tg 3 m, tg 16 m) (n = 12, ANOVA followed by a TUKEY test, **p < 0.01, ***p < 0.001.) and total Aβ detection by immunoprecipitation/western blot and subsequent quantification by densitometry (n = 3, Students t-test, *p < 0.05). (**C**) Quantification of CD11b positive, activated microglia (see insert, arrows) and GFAP positive astrocytes in the hippocampus (HC, open bar) and frontal cortex (FC, filled bars) (n = 12, ANOVA followed by a TUKEY test, *p < 0.05, **p < 0.01,***p < 0.001).

Brain amyloid plaque load was determined in APP [V717I] mice and completely in line with previous studies [6,7]. Amyloid plaques were undetectable by Thioflavin-S or Aβ immunostaining in brains of APP [V717I] mice at 3 months of age but were abundantly present in 16 month old transgenic mice (Fig. 1A). By western blotting, amyloid peptides were evidently detected in brains of APP [717I] mice at both ages (Fig. 1B). In parallel, age-dependent inflammatory changes were assessed in the frontal cortex and hippocampus by immunohistochemistry for CD11b and GFAP, as markers for microglial and astrocytic activation, respectively. In 3 month old wild type controls, clustered CD11b was undetectable but labelled uniformly distributed ramified microglia (Figure 1A). In contrast, brains of APP [V717I] mice showed a focally activated CD11b immunostaining already at 3 months. Microglial morphology identified different activation states, but only round or oval appearing cells were quantified and counted as being "activated" in the hippocampus and frontal cortex (Figure 1A, see insert). In APP [V717I] of 16 months, an even more pronounced excess of activated microglia was obvious in both brain areas (Figure 1B). In keeping with microglial activation, cortical GFAP-immunostaining was practically absent in non-transgenic control mice at 3 months (data not shown), whereas APP [V717I] transgenic mice had randomly distributed foci of astrocytes strongly expressing GFAP within the cortex and hippocampus at that age. While the majority of GFAP-positive foci appeared to be randomly distributed within the cortex and hippocampus, some of these GFAP postive foci were found to surround brain vessels. Quantification of GFAP-positive cells (Figure 1B) demonstrated an even greater increase in the number of activated astrocytes at 16 months compared to age-matched non transgenic mice.

**Figure 2:**
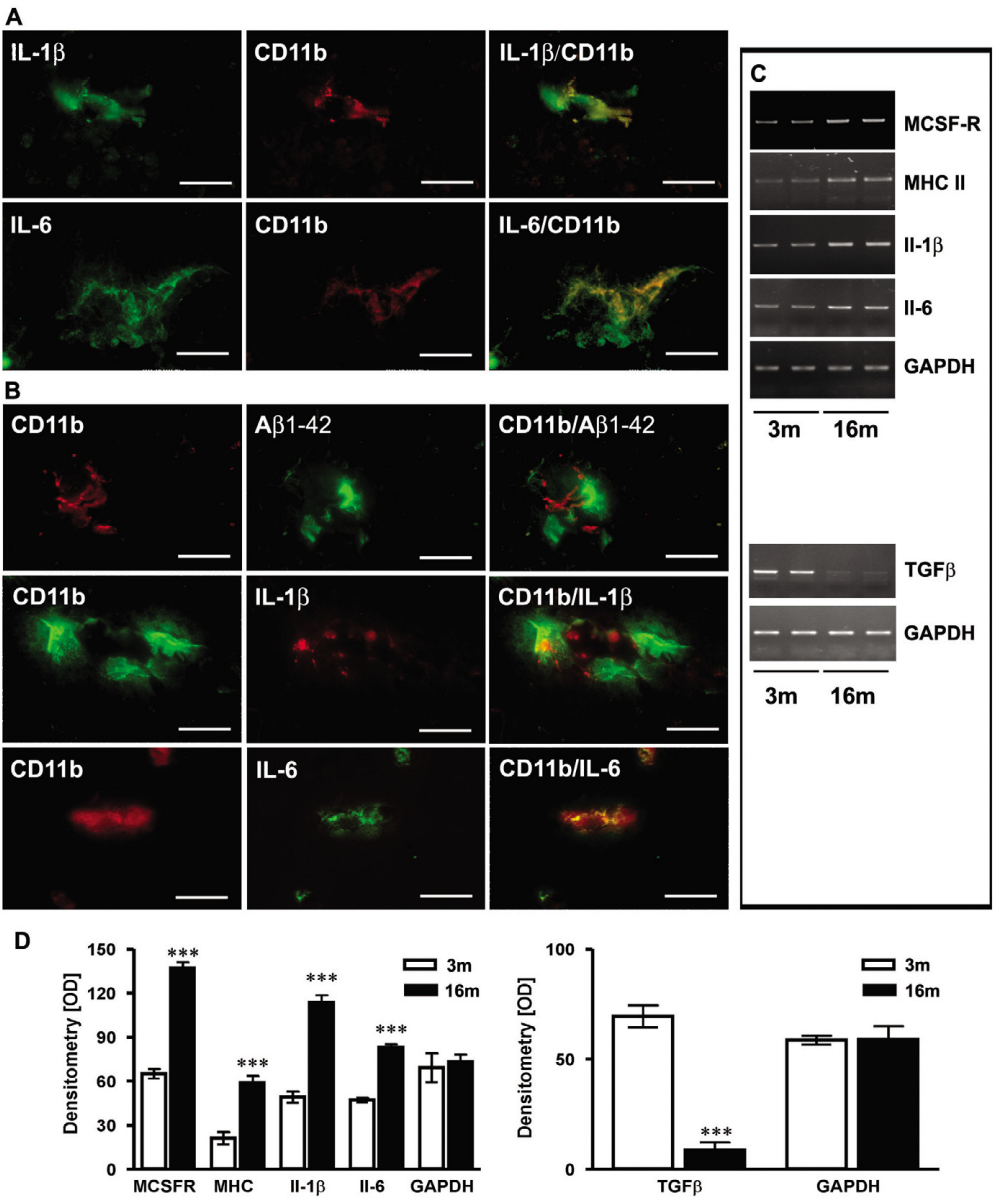
**Characterisation of microglial inflammation. **(**A**) Representative confocal immunohistochemistry of APP [V717I] mice revealed that Il-1β and Il-6 colocalized with activated CD11b-positive microglial cells at 3 month. (**B**) At 16 month CD11b positive cells were almost exculsively detected in close proximity to Aβ1–42 positive plaques. At this time point, CD11b-positive and plaque associated microglia were also found to be colocalized with Il-1β and Il-6. (**C**) RT-PCR analysis was performed with frontal cortex brain lysates and is being displayed from two single animals at each age (3 and 16 month) for Il-1β, Il-6, MCSF-R, MHCII and TGFβ-1 and showed increased gene transcription at 16 months. (**D**) Densitometry of PCR products of APP [V717I] mice at 3 (open bars) and 16 months (filled bars) for the indicated inflammatory molecule. RT-PCR for GAPDH served as control. (n = 6, ANOVA followed by a TUKEY test, ***p < 0.001. Bar graphs in A-B are = 25 μm).

Confocal analysis of immunostaining for CD11b in combination with Il-1β (Figure 2A) or Il-6 at (Figure 2A) at 3 month demonstrated that microglia already produced both cytokines in young APP [V717I] transgenics. Similar results were obtained by double staining for CD11b and MHCII or MCSF-R (not shown). In brains of 16 month old APP [V717I] transgenic mice, Cd11b positive and activated microglia cells were predominantly associated with amyloid plaques as revealed by co-staining with Aβ1–42 (Figure 2B). Further analysis demonstrated that these microglial cells also expressed Il-1β, Il-6 (Figure 2B), MCSF-R and MHC II (not shown). The mRNA coding for Il-1β, Il-6, MHC II and MCSF-R were already detectable in frontal cortex brain lysates of 3 month old APP [V717I] transgenic mice, while absent in non-transgenics (data not shown) and most significantly increased in the brain of old APP [V717I] transgenic mice at 16 months (Figure 2C). Several other cytokines, i.e. tumor necrosis factor alpha, interferon gamma, interleukin-10 and interleukin-4 were undetectable at either age (results not shown). In contrast, TGFβ-1 mRNA levels showed an inversed pattern with signifcantly decreased levels in the brain of 16 month old, relative to young APP [V717I] transgenic mice (Figure 2C).

**Figure 3:**
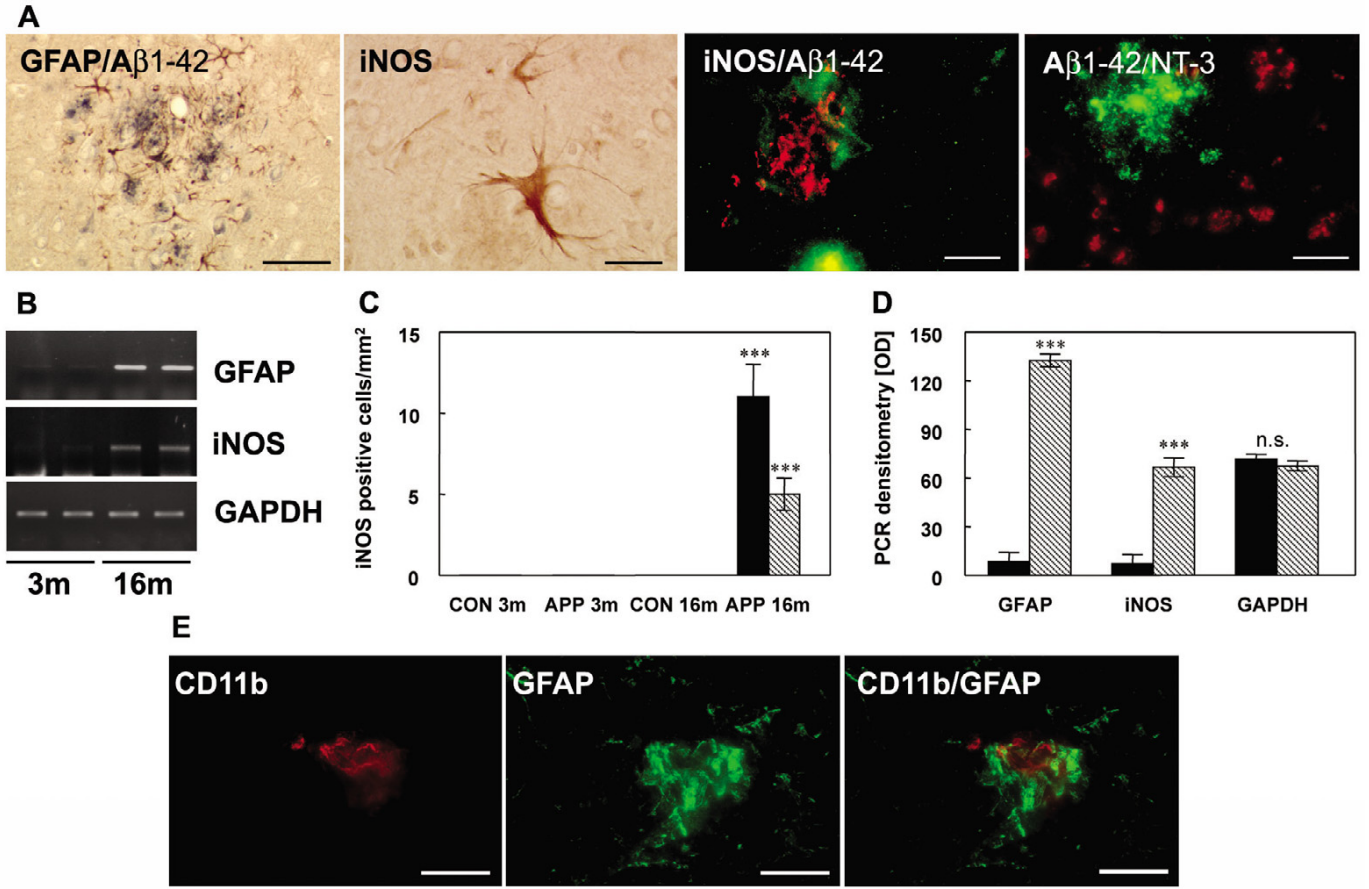
**Astrocytic iNOS expression and plaque associated nitrotyrosine. **(**A**) Costaining of Aβ1–42 and GFAP at 16 months detected activated astrocytes nearby Aβ plaques. Astrocytic iNOS and confocal staining of iNOS (red) and Aβ1–42 (green) or nitrotyrosine (red) and Aβ1–42 (green). (**B**) RT-PCR for GFAP and iNOS in APP [V717I] mice at 3 (3 m) and 16 months (16 m) of age. (**C**) Quantification of iNOS-positive astrocytes in the hippocampus (HC, black bar) and frontal cortex (FC, hatched bars) of APP transgenic mice (tg) and wild type controls (wt) at 3 and 16 months. (**D**) Densitometry of GFAP, iNOS and GAPDH mRNA from APPV [7171I] mice at 3 (black bars) and 16 months (hatched bars). (**E**) Confocal staining of CD11b positive microglia and GFAP labelled astrocytes showed that both cells were located in close neighbouring in APP [V717I] mice at 3 month of age. (n = 6, ANOVA followed by a TUKEY test, n.s. = non significant, ***p < 0.001). Bar graph = 50 μm.

Analysis of astroglial activation by double staining for Aβ1–42 and GFAP demonstrated that GFAP-positive cells were mostly located around amyloid plaques in the brains of aged transgenic mice (Figure 3A). At this age a subset of plaque-associated astrocytes was immunopositive for iNOS in both the hippocampus and the frontal cortex (Figure 3B, C). Confocal staining for GFAP and iNOS confirmed that iNOS positive cells were astrocytes (not shown), and demonstrated their close spatial relation to amyloid plaques (Figure 3A). Additionally, co-staining for nitrotyrosine and Aβ revealed an increased NO-dependent peroxynitrite generation in close proximity to the amyloid plaques (Figure 3A). This result was paralleled by increased iNOS and GFAP mRNA levels in brain of 16 month old APP [V717I] mice (Figure 3B, D). In brains of non-transgenic mice, the iNOS mRNA was not detectable (data not shown). Remarkebly, activated microglial and astrocytic cells were colocalized as demonstrated by double staining for CD11b and GFAP, already in the brain of young APP [V717I] mice, suggesting the formation of inflammatory foci in both brain regions evaluated (not shown).

**Figure 4:**
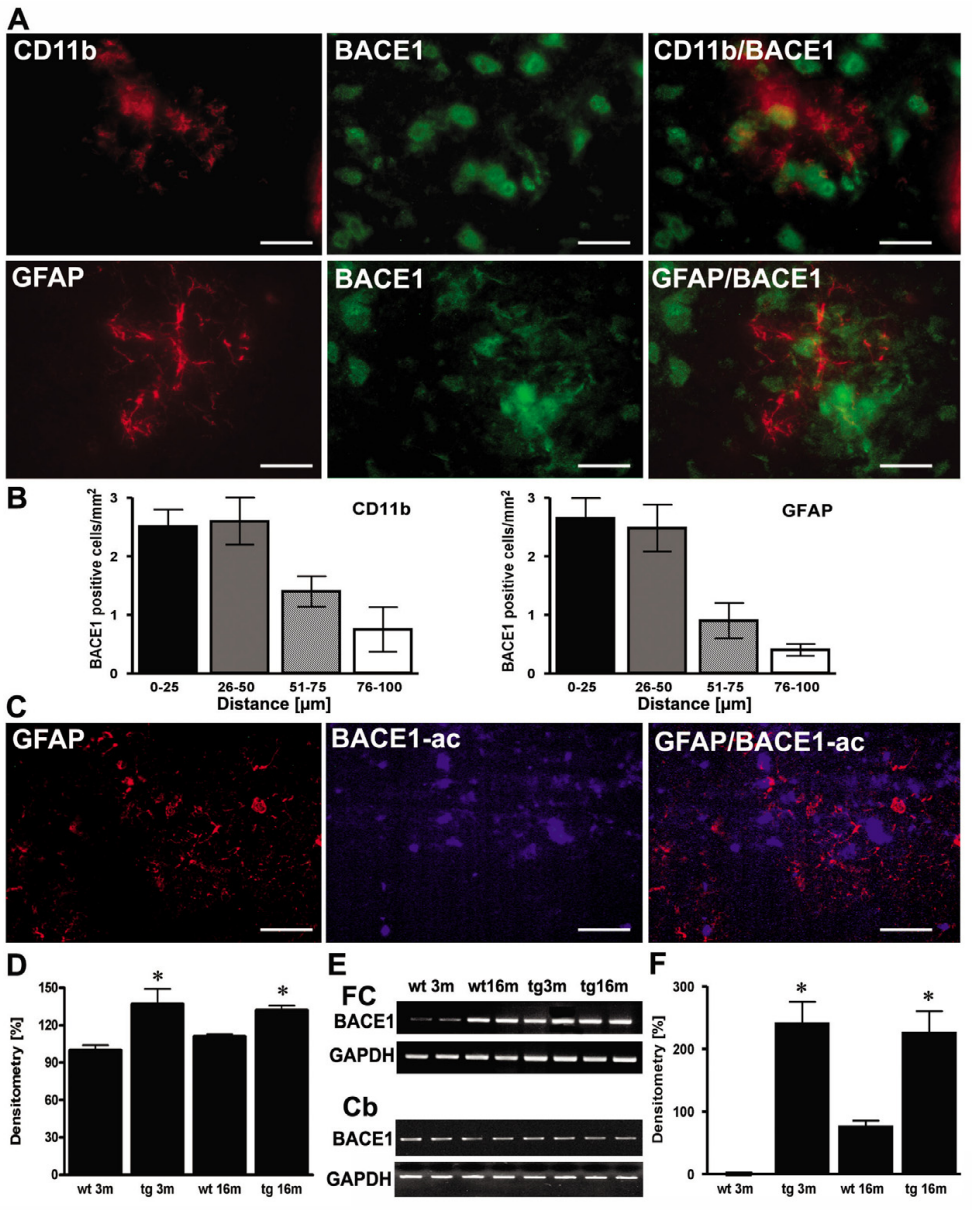
**Sites of focal and early inflammation show BACE1 upregulation in neurons. **(**A**) Representative confocal immunostaining of CD11b positive microglia and BACE1 and GFAP and BACE1 in 3 month old APP transgenics showed that BACE positive neurons were found close to focally activated microglia cells in 3 month old APP [V717I] mice. (**B**) Quantitation of the number of BACE1 positive cells in relation to the distance to CD11b or GFAP positive cells. (**C**) Representative image of focal GFAP expression, BACE1 activity and overlay in APP [V717I] mice at 3 m of age. (**D**) Measurement of BACE1-activity was calculated as percentage of 3 month old controls (wt 3 m) and showed that enzyme activity was already elevated in APP [V717I] mice at 3 month (tg 3 m) (n = 5, ANOVA followed by a TUKEY test, *p < 0.05). (**E**) RT-PCR detection of BACE1 mRNA levels of cortical (frontal cortex, FC) and cerebellar (Cb) lysates from wild type controls (wt), APPV [7171I] (tg) mice at 3 (3 m) and 16 months (16 m). (**F**) Densitometrical analysis and quantitation of BACE1 mRNA levels of frontal cortex lysates of APP [V717I] transgenic and controls at the respective age (n = 6, ANOVA followed by a TUKEY test, *p < 0.05). Bar graphs are = 50 μm for CD11b/neuN and GFAP/BACE and = 25 μm for CD11b/BACE1).

**Figure 5:**
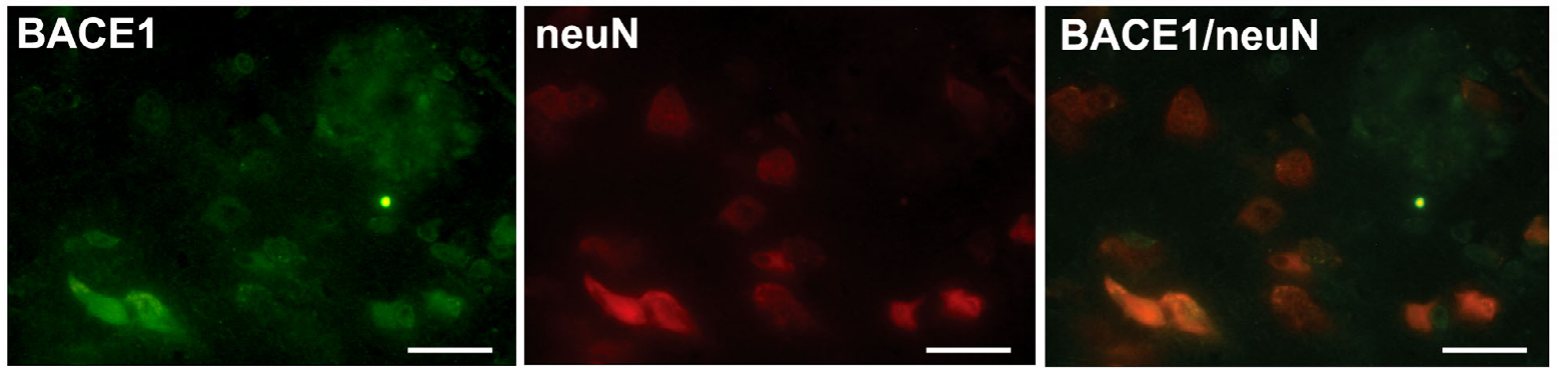
**Expression of BACE1 in neurons in APP [V717I] transgenic mice at 3 month of age. **Representative confocal immunostaining of BACE1 positive cells and neuN positive neurons in the cortex of 3 month old APP [V717I] mice. Bar graphs are = 50 μm for BACE1, neuN and BACE1/neuN.

Since we demonstrated that cytokine stimulated neuronal cells increased production of Aβ by transcriptional BACE1 up-regulation in vitro [18], and the latter cytokines were detectable at sites of early inflammation in young APP [V717I] mice, we next analysed whether early inflammatory foci would be accompanied by BACE1 expression. Co-staining for BACE1 and neuN demonstrated that neurons expressed BACE1 in the 3 month old APP [V717I] mice throughout the cortex and hippocampus, confirming a previous observation in another transgenic mouse model [21] (data not shown). Despite the fact that BACE1 was expressed widely, a clear and focal upregulation of neuronal BACE1 immunostaining was observed in brain of APP [V717I] transgenic mice at 3 months of age. Costaining for CD11b and BACE1 or for GFAP and BACE1 showed that the upregulation was predominantly confined to neurons which were located in close proximity to CD11b positive microglia (Fig. 4A). The neuronal nature of BACE expressing cells was further confirmed by confocal immunostaining for the neuronal marker neuN and BACE 1 (Figure 5). Subsequent quantification of BACE1 expressing neurons confirmed that the highest number of BACE1 positive cells were in close distance to both CD11b and GFAP activated micro- and astroglial cells (Figure 4B).

*In situ *fluorescence detection of BACE1 activity revealed that sites of increased BACE1 activation colocalised to GFAP positive and activated astrocytes (Figure 4C). Addition of a previously described BACE1 inhibitor served as control [20] and abrogated the signal (data not shown). Quantitative determination of BACE1 activity from cortical lysates showed that BACE1 enzyme activity was significantly increased in brains of 3 month old APP [V717I] mice when compared to controls and did not further increase at 16 month (Figure 4D). This phenomenon was paralleled by increased BACE1 mRNA levels in the frontal cortex, whereas at the same time cerebellar BACE1 mRNA levels did not reveal any significant regulation (Figure 4E, F). Combined, these data indicate the inflammation-associated increase in BACE1 levels in brain of young, 3 month old APP [V717I] mice compared to age-matched non-transgenic mice.

## Discussion

In AD, the deposition of amyloid peptides and neurofibrillary tangles are invariably associated with an inflammatory component, mainly characterized by activated microglial cells and astrocytes. Aβ peptides and secreted APPs are potent activators of glia cells [22]. Once activated, micro- and astroglia release a variety of cytokines, chemokines and free radical oxygen species, which can contribute to neuronal dysfunction and death. In addition, some specified glia-derived cytokines may also increase Aβ generation [23]. The finding that several cytokines increase total and fibrillogenic Aβ by transcriptional upregulation of BACE1 mRNA, protein and activity levels [18] suggests a morbid feedback mechanism by which neurodegenerative and neuroinflammatory mechanisms interact. Activated microglia may, however, play a dual role in AD, since clearance of Aβ through phagocytosis [24] may be advantageous. To define the active contribution of inflammation in AD, experimental animal models are needed that recapitulate both the neurodegenerative and the inflammatory components of the disease.

Whereas transgenic mouse models are widely used to study APP processing, only a limited number of studies has addressed neuroinflammation in these animals, yielding in part controversial results. Thus, APP695 transgenic mice aged 2 to14 month failed to reveal mRNA for several cytokines including Il-1α/β, Il-6, Il-10, Il-12 and IFNγ by ribonuclease protection assay [25]. In the same study, however, Il-1β-positive astrocytes were detected in close proximity to amyloid deposits in older mice, whereas immunohistochemistry for TNFα, Il-1α, Il-6, and MCP-1 was negative. In contrast, TNFα mRNA was evident as early as 6 month [26] and IFNγ and Il-12 mRNA and protein was detected by in situ hybridization and immunohistochemistry in 9 month old APP695 transgenic mice [27]. Moreover, Il-1β, TNFα and Il-10 was detected by immunohistochemistry in animals at 12 and 13 month of age [28,29]. The differences reported in the same strain of APP transgenic mice are likely to be caused by different techniques employed and demonstrate the difficulties encountered in assessing inflammatory changes in the brain of this mouse model.

In contrast to these studies, the present work revealed a significant increase in focally activated microglia cells expressing cytokines such as Il-1β and Il-6 already at 3 months, which was paralleled by mRNA levels for Il-1β, Il-6, MHC II and MCSF-R. At this age, these APP [V717I] mice do not yet deposit amyloidogenic Aβ peptides as verified by the complete absence of immunopositive and Thioflavin-S-positive plaques, confirming previous results [6,7]. Microglial foci seemed to be randomly distributed in the cortex and hippocampus of 3 month old APP transgenic mice. However, since total levels of Aβ were already detectable at this age and soluble fragments also act as potent stimulators of microglial cytokine secretion [30], soluble Aβ along with secreted APP [22] may cause this early microglial activation long before amyloidogenic fragments deposit.

It is most interesting to note that the APP [V717I] transgenic mice develop cognitive impairment, decreased long-term potentiation (LTP) and neophobia already at 3 month of age [6]. Importantly, this phenomenon was not correlated with the actual APP isoform expressed nor with the levels of a single APP metabolite [6]. Because cytokines including Il-1β and Il-6 directly impair neuronal function and suppress hippocampal LTP [31,32] the current data allow us to propose that early and focal inflammatory events contribute to neuronal dysfunction at this age. The foci contain moreover all the ingredients needed to generate amyloid peptides and are tentatively identified as "birth-places" of amyloid plaques, resulting from a viscious circle instilled by amyloid peptides and immuno-modulatory factors.

Focal microglia activation was surrounded by GFAP-positive astrocytes in young mice, but GFAP mRNA levels were almost undetectable at 3 month [33]. However, GFAP and iNOS mRNA levels became detectable in transgenic mice at 16 month indicating strong astrocytic activation. Increased GFAP mRNA levels were paralleled by increased numbers of GFAP-positive and iNOS expressing astrocytes. Importantly, iNOS mRNA and protein levels were undetectable in non-transgenic controls and in young APP [V717I] mice. In addition, expression of iNOS in Aβ plaque-associated astrocytes was paralleled by an increase of nitrotyrosine staining indicating enhanced generation of NO dependent peroxynitrite. Because iNOS expression and increased nitrotyrosine staining has been attributed to AD before [34,35], APP [V717I] mice also resemble this aspect of neurodegeneration-induced glial inflammation. In contrast to other cytokines, TGFβ-1 mRNA levels decreased in ageing APP [V717I] mouse brain. Because TGFβ-1 acts mostly as an anti-inflammatory cytokine, age-related loss may facilitate the observed neuroinflammation.

Since we showed most recently that several cytokines, alone and potently in concert, increased Aβ40 and Aβ42 levels by transcriptional upregulation of BACE1 [18], we tested and demonstrated that microglia-derived cytokine generation in early inflammatory foci was accompanied by BACE1 upregulation in brain of young APP [V717I] transgenic mice. At 3 month of age, BACE1 expression was exclusively restricted to neurons confirming studies by in sity hybridisation in Tg2576 and PDAPP mice [36,37]. However, in both major brain regions, i.e. hippocampus and cortex, the increased neuronal BACE1 expression appeared to be clustered. Costaining with CD11b or GFAP and subsequent quantification demonstrated that neuronal BACE1 expression was upregulated in close proximity to activated microglia and astrocytes. Irrespective whether inflammatory mediators or β-site APP-cleavage derived products occur first, the early and focal presence of immunoactive microglia, cytokines and BACE-expressing neurons strongly points to an interaction between neurodegenerative and neuroinflammatory events. In keeping with this finding, hippocampal BACE1 mRNA levels were significantly increased in 3 month old APP [V717I] transgenics compared to non-transgenic mice and this phenomenon was paralleled by strongly increased BACE1 enzymatic activity as determined from brain lysates. In old mice BACE1 expression was also detected in activated astrocytes as observed in Tg2576 mice, but not different from non-transgenic mice [21]. However, the fact that the observed changes of BACE1 RNA levels are higher than those observed for activity; parallels our previous in vitro results [18] and may just indicate that BACE1 activity is regulated not only by gene transcription but at multiple steps thereafter. Interestingly, BACE1 mRNA levels did not significantly change between 3 and 16 month of age in APP [V717I] transgenics. While the current study did not identify the underlying reason of this phenomenon, It can be hypothesized, that irregardeless of higher levels of inflammatory mediators present at 16 month, it is possible that (i) the total spectrum of pro-inflammatory and antiinflammatory mediators is more or equally permissive for BACE 1 upregulation at a very early age, or (ii) the increase of pro-inflammatory cytokines at later ages are accompanied by counteracting anti-inflammatory molecules, resulting in a similar netto induction of BACE1. In addition, several other mechanisms may account for the almost equal levels of BACE1 mRNA at 3 and 16 month of age including a desensitized transcriptional activation, a rebalance between production and degradation of the BACE1 transcript a later age or a lower contribution from disease affected neurons in the close proximity to amyloid plaques.

## Conclusion

APP [V717I] transgenic mice do not only model the late amyloid pathology in parenchym and vasculature as in AD patients, but exhibit also many inflammatory parameters ascribed to the AD pathology. The early and focal neuro-inflammatory changes are demonstrated here to be parallelled closely by upregulated neuronal BACE1 mRNA and protein expression and by increased BACE1 enzyme activity, already in young APP transgenic mice, before any amyloid deposition is evident. The vicious cycle of APP proteolytic cleavage giving rise to soluble and amyloidogenic immunostimulators, causing microglial activation, cytokine generation, is closed by the upregulation of BACE1, ultimately enhancing further APP processing. This cycle appears to operate locally, in focal nidi of disease that could represent the birthplaces of amyloid plaques, already present early in the disease process in brain of young APP transgenic mice.

## Competing interests

The author(s) declare that they have no competing interests.

## Authors' contributions

Michael Heneka: conception and design, immunostaining, data aquisition, interpretation, article writing

Magdalena Sastre: conception, BACE1 measurements

Lucia Dumitrescu-Ozimek: Immunostaining, data aquisition

Ilse Dewachter: amyloid determination

Jochen Walter: BACE1 measurements in situ,

Thomas Klockgether: conception and design,

Fred van Leuven: conception and design, data analysis and interpretation
